# Application of generalized linear models to investigate functional synaptic coupling and synchrony in an animal model of schizophrenia

**DOI:** 10.1186/1471-2202-16-S1-P175

**Published:** 2015-12-18

**Authors:** Jennifer L Zick, Rachael K Blackman, Matthew V Chafee, Theoden I Netoff

**Affiliations:** 1 Graduate Program in Neuroscience, University of Minnesota, Minneapolis, MN 55455 USA; 2 Medical Scientist Training Program (MD/PhD), University of Minnesota, Minneapolis, MN 55455 USA; 3 Brain Sciences Center, VA Medical Center, Minneapolis, MN 55417 USA; 4 Department of Biomedical Engineering, University of Minnesota, Minneapolis, MN 55455 USA

## 

Current hypotheses about the pathophysiology of schizophrenia suggest that the disease results from disordered connectivity in the brain. Human functional imaging studies have lent support to this idea by demonstrating reduced temporal correlations between cortical areas in schizophrenic patients [1]; however, functional evidence for disconnectivity at the synaptic level is limited. Here we describe a study in which we applied computational modeling techniques to assess functional connectivity between spiking neurons in a pharmacological nonhuman primate model of schizophrenia [2]. Neural data were obtained from multielectrode recording arrays inserted into the parietal and prefrontal cortices of macaque monkeys while the animals performed a cognitive control task that measures a specific cognitive impairment in human patients with schizophrenia. Phencyclidine, an NMDA receptor (NMDAR) antagonist with well-described schizomimetic properties [3], was administered systemically on alternating days with injections of saline.

To characterize changes in synaptic communication between neurons in the disease state, we evaluated timing relationships in spike trains of simultaneously recorded neurons. We employed a Generalized Linear Model (GLM) approach [4] to infer patterns of functional connectivity between neurons. The result of the GLM fit is a set of coupling functions that estimate the change in the probability of a spike in one neuron in response to a spike in another simultaneously recorded neuron. Unlike traditional cross-correlation approaches used for inferring functional connectivity, GLMs parse out the proportion of variance in spike timing attributable to synaptic interactions between neurons from other sources of variance in spike timing such as stimulus input, the intrinsic spike patterns of the cells, and the effects of other simultaneously recorded cells in the network [4]. After identifying functionally coupled pairs of neurons using GLMs, we sought to evaluate the effects of phencyclidine on functional coupling and synchrony between coupled cells. We found a dramatic decrease in the proportion of cells that were functionally coupled in the prefrontal cortex in the phencyclidine condition as compared to the control condition. In order to investigate hypothesized alterations in spike timing in the disease state, we identified the distribution of interspike intervals between putative pre- and postsynaptic neurons. In our analysis we found a prominent "zero-lag" peak representing a large number of coincident action potentials between coupled cells in the saline condition, but not in the phencyclidine condition. This suggests a change in the timing of action potentials which was apparent in the absence of a change in firing rates, precluding overall decreased activity as an explanation for reduced synchrony. In summary, these results suggest that phencyclidine induces a functional disconnection between synaptically coupled cortical neurons which may be related to a Hebbian reduction in synaptic strength resulting from desynchronization of spiking activity.
